# Effectiveness of oral care interventions on malodour in dogs

**DOI:** 10.1186/s12917-022-03267-8

**Published:** 2022-05-05

**Authors:** Julie M. Croft, Krusha V. Patel, Taichi Inui, Avika Ruparell, Ruth Staunton, Lucy J. Holcombe

**Affiliations:** Waltham Petcare Science Institute, Melton Mowbray, Leicestershire, LE14 4RT UK

**Keywords:** Oral malodour, Volatile sulphur compounds, Oral care interventions, Dogs, VSC-producing bacteria

## Abstract

**Background:**

Oral malodour is identified by pet owners as an unpleasant inconvenience, but they may not recognise this likely indicates underlying disease. The primary cause of oral malodour relates to the presence of bacteria in the oral cavity often associated with gingivitis and periodontitis. The purpose of this study was to determine the effect of feeding two oral care chews with different textural properties on oral malodour and the proportion of bacterial species involved in the production of volatile sulphur compounds (VSCs).

**Methods:**

Fourteen dogs (9 Petit Basset Griffon Vendéen (PBGV) and 5 Beagle dogs**)** participated in the randomised cross-over study for a total of 14 weeks. The cohort was divided into four groups with each exposed to a different intervention per week: chew A, chew B, tooth brushing control or a no intervention control. An induced malodour method was used to assess VSCs in breath samples using a portable gas chromatograph (OralChroma™). Microbiological samples (supragingival plaque and tongue coating scrapes) were analysed for VSC-producing bacteria using Oral Hydrogen Sulfide agar with lead acetate.

**Results:**

VSCs were detected in the dogs’ breath samples and levels of hydrogen sulphide and methyl mercaptan were found to be reduced following an intervention. Chew B significantly reduced the levels of both hydrogen sulphide (*p* < 0.001) and methyl mercaptan (*p* < 0.05) compared to no intervention. Reductions in methyl mercaptan were also observed for chew A and tooth brushing but these were not statistically significant. When compared to no intervention, all interventions significantly reduced the total bacterial load and VSC producing bacterial load in plaque (*p* < 0.001). For tongue samples, only chew B significantly reduced the total bacterial load and VSC-producing bacterial load (*p* < 0.001) compared to no intervention.

**Conclusions:**

By inducing oral malodour and subsequently applying the one-time interventions, significant reductions in the levels of VSCs were observed. The use of oral care chews texturally designed to deliver a deep, all-round cleaning action can be particularly effective at managing oral malodour in dogs, likely through an enhanced ability to remove bacteria.

## Background

The aetiology of oral malodour, otherwise known as halitosis, is primarily attributed to products generated from bacterial metabolism of sulphur-containing amino acids, such as cysteine and methionine. In the human oral cavity over 700 compounds have been identified such as indole, skatole, and volatile sulphur compounds (VSCs) [1]. The concentration of VSCs is strongly correlated to the degree of oral malodour, with the lowest value of human odour detection correlating with methyl mercaptan (CH_3_SH), followed by hydrogen sulphide (H_2_S) and dimethyl sulphide ((CH_3_)_2_S). These three substances are considered the main contributors to the unpleasant smell in the human and canine mouth [2, 3].

The majority of dog owners perceive the condition to be a cosmetic problem. However, increasing evidence from the human literature suggests that even extremely low concentrations of many of these compounds are highly toxic to tissues and their presence is associated with periodontal disease [2, 4, 5]. Periodontal disease is one of the most common diseases seen in small animal practices [6, 7]. It is a progressive, cyclical, inflammatory condition of the supporting structures of the teeth and the main cause of dental disease and early tooth loss in dogs. Oral malodour is one of the first signs of periodontitis noticed by pet owners, it therefore represents an important indicator of a potential underlying issue. The association between oral malodour, VSC production and periodontal disease in dogs has been summarised in a review [8]. The periodontal disease process is thought to be more pronounced in dogs due to the alkaline oral environment, a result of the salivary pH being approximately 8.5 [9], which is substantially higher than the human pH normal range of 6.2-7.6 [10]. An oral environment which has a high pH can also favour the growth of bacterial species known to be associated with periodontal disease such as *Actinomyces,* and *Porphyromonas* [11]. VSCs have been shown to increase the permeability of the oral mucosa allowing substances such as endotoxins and prostaglandins to penetrate the tissue barrier [12]. This is of paramount importance in the development of periodontitis, initiating an inflammatory response and ultimately leading to the exposure of the underlying connective tissue to periodontal pathogenic compounds [8].

The canine oral cavity harbours a rich, diverse bacterial community that is widely divergent from that of humans [13, 14]. Literature relating to canine oral malodour microbiology is scarce, meaning correlations are often based on evidence from human studies. A large consort of bacteria within the human oral cavity are thought to be involved in the production of VSCs [15]. The most commonly identified microbes producing VSCs are Gram-negative anaerobes, which include *Fusobacterium* sp, *Treponema* sp, *Porphyromonas* sp and *Bacteroides*, and Gram-positive genus *Peptostreptococcus* [16]. Other insights from human oral malodour studies suggest the location of such species is important in the development of the condition; key sites include the posterior dorsal tongue, gingival crevices, periodontal pockets and saliva [17, 18]. In a recent study of 14 Labrador retrievers, the same genera of bacteria were identified on the tongue, in supragingival plaque and saliva, suggesting they may also contribute to oral malodour in dogs [19]. In addition, these genera of bacteria have been associated with periodontal disease in both humans and companion dogs [20, 21].

Several measurement methods have been used to assess VSC levels in human and animal models. Organoleptic assessment requires a trained human sensory panel to evaluate the intensity of odour from samples presented to the nose. Depending on the level of discrimination, this can be either qualitative or quantitative analyses. Panellists can be trained to distinguish between malodorous chemical compounds, as well as to rank them according to levels of perceived offensiveness [22]. The application of scientific appliances capable of detecting sulphur, such as a Halimeter^®^ or an OralChroma™, can provide more quantitative outputs [22]. The Halimeter^®^ contains a gas sensor that detects a range of compounds, H_2_S, CH_3_SH, other thiols, and (CH_3_)_2_S, generating a combined gas measure. The OralChroma™ is a more sensitive, portable, gas chromatograph machine which can discriminate samples via three VSCs (H_2_S, CH_3_SH and (CH_3_)_2_S) to deliver individual, quantitative gas measurements in real time. To ensure gas values are within the detection limits of the appliances, supplementation with cysteine and methionine amino acids to the diet can be used to induce the production of H_2_S and CH_3_SH [23, 24]. *Peptostreptococcus, Eubacterium, Bacteroides* and *Fusobacterium* sp are particularly active in the production of H_2_S from L-cysteine, whilst some *Fusobacterium, Bacteroides, Porphyromonas* and *Eubacterium* sp form CH_3_SH from L-methionine [25].

Good oral hygiene practice is recognised as a means to manage malodour in dogs [26] and poor oral care is the most significant risk factor in the development of periodontal disease. A number of studies have shown that the accumulation of dental plaque on dogs’ teeth is often associated with the severity of gingivitis and periodontitis [14, 20, 27, 28]. Many owners do not regularly brush their dog’s teeth or arrange for routine cleaning by a veterinary professional [29, 30]. However, even with effective tooth brushing, plaque removal on the lingual and palatal surfaces of teeth or in grooves can be difficult. Regular incorporation of dental chews into a dog’s feeding regime has the potential to promote periodontal health [31] as well as reduce oral malodour. A study to assess the benefits of feeding a daily dental chew in dogs found statistically significant reductions in plaque and calculus accumulation and oral malodour while improving gingival indices [32]. Another study investigated the effect of a vegetable dental chew and showed daily administration decreased halitosis as well as significantly reducing gingivitis, plaque and calculus accumulation [33]. Chews, such as these may therefore play a significant role in the long-term improvement of canine oral health. Malodour assessment in both studies was performed using the Halimeter^®^ which, as mentioned above, cannot distinguish between the three key component gases. The present study evaluated an effect of the single instance of an oral intervention on VSCs detected in canine breath using an OralChroma™ device, which is considered a highly sensitive alternative. Additionally, plaque and tongue sample scrapings were cultured on a selective media to determine the bacterial load of VSC-producing bacteria and to establish if there was a correlation between these bacteria and the VSCs detected in breath.

## Results

Nine Petit Basset Griffon Vendéen (PBGV) and five Beagle dogs were recruited and completed the study. In total, 159 breath samples were measured using the OralChroma™, and 159 plaque and 159 tongue scrape samples were collected and analysed for VSC-producing bacteria. Chews were consumed on all occasions when presented to the dogs. A minimum 2 mins tooth brushing was not achieved on 13/159 occasions, however an average of 2.59 mins and median of 2.05 mins was achieved.

### VSC detection in canine breath

**Fig. 1:**
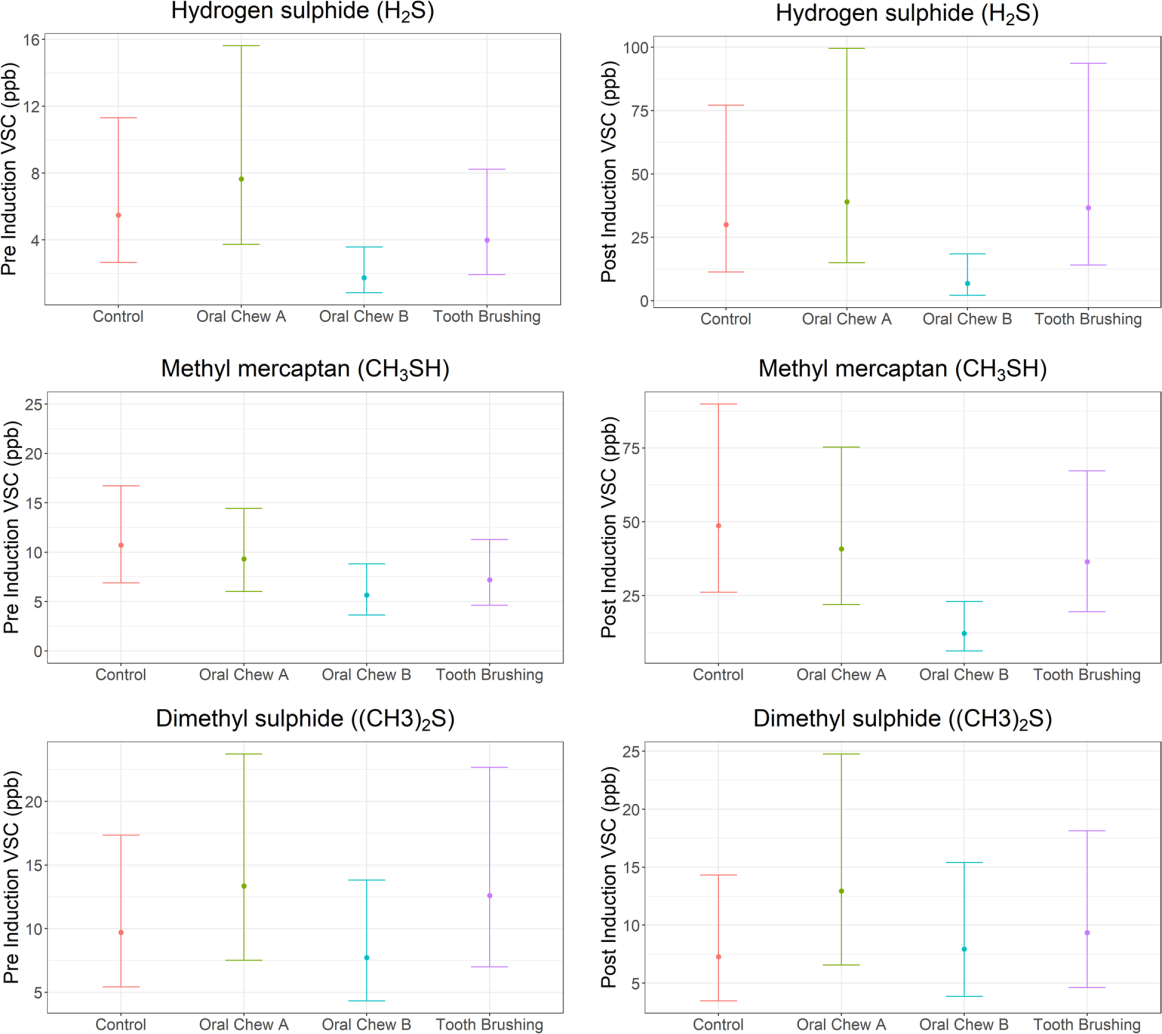
Pre and Post-induction estimated means between interventions for hydrogen sulphide (H_2_S), methyl mercaptan (CH_3_SH)) and dimethyl sulphide ((CH_3_)_2_S)), with 95% confidence intervals

VSCs, hydrogen sulphide (H_2_S), methyl mercaptan (CH_3_SH) and dimethyl sulphide ((CH_3_)_2_S) were successfully detected in canine breath samples using the OralChroma™. Pre-treatment values for all 3 VSC’s detected were near the limit of detection for the OralChroma™, however following a cysteine induction method to induce malodour, H_2_S and CH_3_SH levels increased, while no difference was detected for (CH_3_)_2_S. Mean estimates were calculated for each intervention pre- and post-induction (Table 1). Significant differences were observed between interventions for both H_2_S and CH_3_SH post induction with the feeding of oral care chew B compared to the no intervention control (*p* < 0.01 and *p* < 0.001). (CH_3_)_2_S levels did not significantly differ between treatment and the no intervention control. Also, no significant differences were detected for H_2_S or CH_3_SH for oral care chew A or tooth brushing when compared to the control. The estimated means are shown in (Table 1, Fig. 1) and observed differences (Table 2, Fig. 1) for levels of VSCs detected between each intervention and the control.

**Table 1:** Estimated means and 95% confidence intervals pre and post-induction for each of the Volatile sulphur compounds: (*VSCs*), hydrogen sulphide (*H*_*2*_*S*), methyl mercaptan (*CH*_*3*_*SH*) and dimethyl sulphide ((*CH*_*3*_)_*2*_*S*) in parts per billion (ppb)

		Pre- induction (ppb)			Post- induction (ppb)		
VSC	Intervention	Estimated means	95% lower confidence limit	95% upper confidence limit	Estimated means	95% lower confidence limit	95% upper confidence limit
H_2_S	No Intervention	5.5	2.7	11.3	30.0	11.3	77.1
H_2_S	Chew B	1.7	0.8	3.6	6.8	2.1	18.5
H_2_S	Tooth brushing	4.0	1.9	8.2	36.7	14.0	93.7
H_2_S	Chew A	7.6	3.7	15.6	39.0	14.9	99.6
CH_3_SH	No Intervention	10.7	6.9	16.7	48.7	26.2	89.9
CH_3_SH	Chew B	5.7	3.6	8.8	12.2	6.3	23.0
CH_3_SH	Tooth brushing	7.2	4.6	11.3	36.5	19.6	67.3
CH_3_SH	Chew A	9.3	6.0	14.4	40.9	22.0	75.3
(CH_3_)_2_S	No Intervention	9.7	5.4	17.4	7.3	3.5	14.3
(CH_3_)_2_S	Chew B	7.7	4.3	13.8	7.9	3.9	15.4
(CH_3_)_2_S	Tooth brushing	12.6	7.0	22.7	9.4	4.6	18.1
(CH_3_)_2_S	Chew A	13.4	7.5	23.7	12.9	6.6	24.8

**Table 2:** Pre and post induction estimated differences in fold changes between interventions modelled using a linear mixed effect model 95% confidence intervals and *p* values are reported. For each Volatile sulphur compounds (*VSCs*), hydrogen sulphide (*H*_*2*_*S*), methyl mercaptan (*CH*_*3*_*SH*) and dimethyl sulphide ((*CH*_*3*_)_*2*_*S*)

		Pre- induction				Post- induction			
VSC	Intervention contrast	Estimated fold change	95% lower confidence limit	95% upper confidence limit	***p*** value	Estimated fold change	95% lower confidence limit	95% upper confidence limit	***p*** value
H_2_S	Chew B / No Intervention	0.25	0.09	0.72	**0.01**	0.25	0.09	0.72	**0.01**
H_2_S	Tooth brushing / No Intervention	1.22	0.43	3.48	0.95	1.22	0.43	3.48	0.95
H_2_S	Chew A / No Intervention	1.29	0.45	3.70	0.89	1.29	0.45	3.70	0.89
CH_3_SH	Chew B / No Intervention	0.27	0.16	0.45	**< 0.001**	0.27	0.16	0.45	**< 0.001**
CH_3_SH	Tooth brushing / No Intervention	0.75	0.44	1.29	0.46	0.75	0.44	1.29	0.46
CH_3_SH	Chew A / No Intervention	0.84	0.49	1.44	0.79	0.84	0.49	1.44	0.79
(CH_3_)_2_S	Chew B / No Intervention	1.08	0.53	2.20	0.99	1.08	0.53	2.20	0.99
(CH_3_)_2_S	Tooth brushing / No Intervention	1.25	0.61	2.56	0.80	1.25	0.61	2.56	0.80
(CH_3_)_2_S	Chew A / No Intervention	1.69	0.82	3.45	0.21	1.69	0.82	3.45	0.21

To investigate if the time a dog was exposed to an intervention influenced the levels of VSCs detected, the time taken for each treatment was recorded. Consumption of chew B was found to represent the longest treatment duration with a mean of 12.26 mins (median 11.38), followed by consumption of chew A 3.09 mins (median 3.04) and tooth brushing 2.59 mins (median 2.05). Neither the time dogs spent keeping their muzzles in the mask nor the time they spent consuming amino acid-containing gels showed a correlation to the amount of VSCs produced (data not shown).

### Bacterial culture analysis

**Fig. 2:**
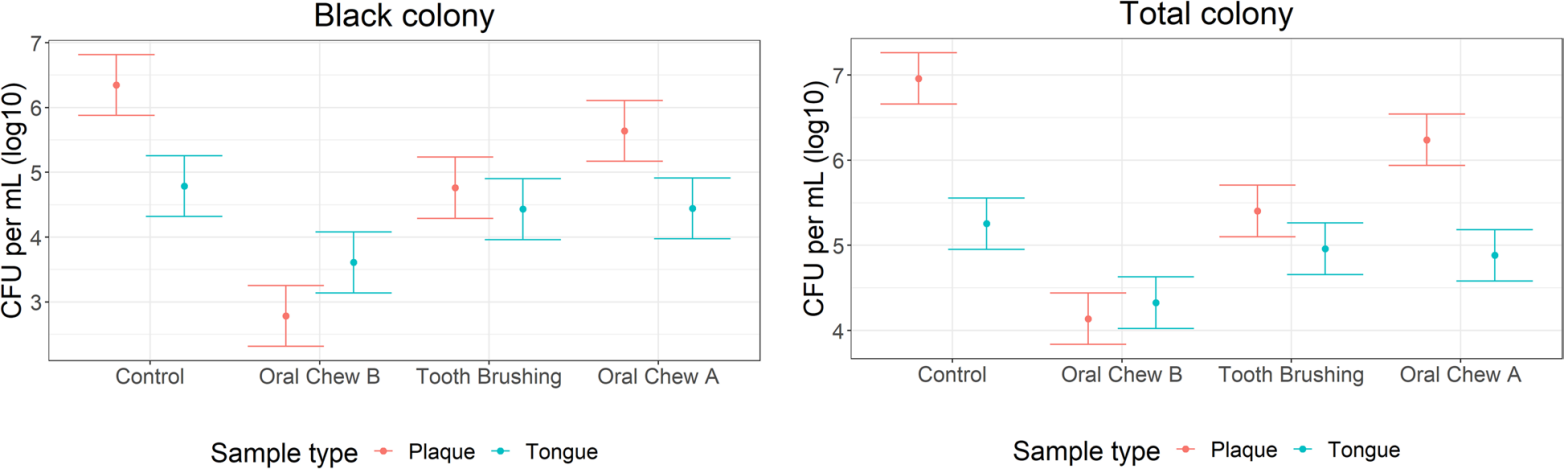
Plaque and tongue total (A) and black (B) colony counts CFU per ml (log_10_) estimated means and 95% confidence intervals

**Fig. 3:**
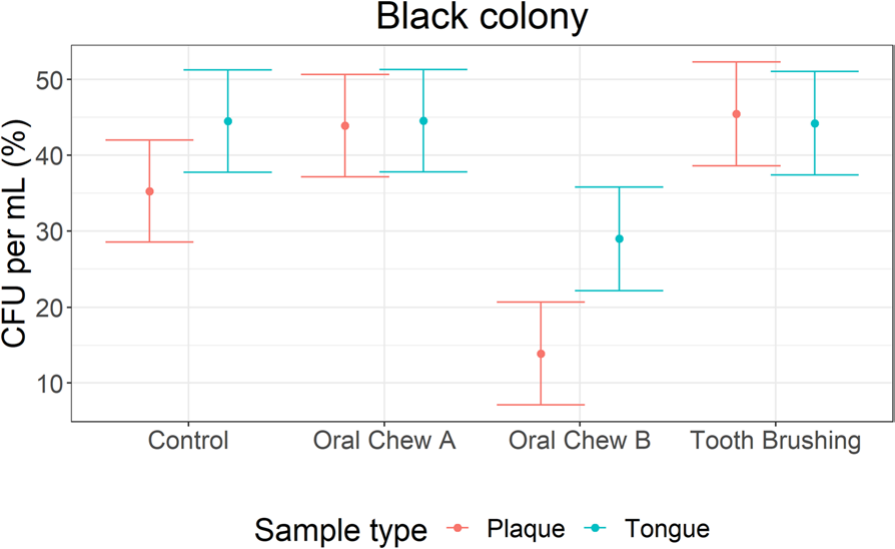
Average percentage of black colonies out of the total numbers for each sample type and intervention, with 95% confidence intervals

Bacteriological analysis using OHO-C agar revealed the presence of VSC-producing bacteria in both plaque and tongue scrape samples. VSC-producing microbes were visualised as dark black colonies, which had produced lead sulphide precipitates on the surfaces of agar plates. Counts of other colony colours (white/cream) combined with black colonies were recorded to estimate the total number of culturable bacteria present. In plaque samples, significant reductions in total colony counts (*p* < 0.001) and black colony counts (*p* < 0.001) were observed for dogs receiving the treatment interventions oral care chew A, oral care chew B and tooth brushing compared to no intervention control. Only dogs exposed to oral care chew B had a significant reduction in total colony counts (*p* < 0.001) and black colony counts (VSC-producing bacteria) (*p* < 0.001) in tongue scrape samples, compared to the no intervention control (Fig. 2A and B). Significant fold changes between the interventions and the control for black colonies were observed for oral care chew A, oral care chew B and tooth brushing (*p* < 0.001) in plaque samples and oral care chew B for tongue samples (p < 0.001) (Table 3). The proportion of black colonies were also calculated and for both plaque and tongue samples, the percentage of black colonies was significantly lower for oral care chew B compared to the control. No significant difference compared to the control was observed for oral care chew A or tooth brushing compared to the control (Fig. 3).

**Table 3:** Estimated fold changes from no-intervention to all other interventions within each sample type for number of black colonies with 95% confidence intervals and *p*-values for significance (bold text)

Contrast	Estimated fold change	95% lower confidence limit	95% upper confidence limit	***p*** value
Plaque: No Intervention / Chew B	3649.1	1624.9	8194.8	**< 0.001**
Plaque: No Intervention / Tooth brushing	38.3	16.9	86.9	**< 0.001**
Plaque: No Intervention / Chew A	5.1	2.3	11.2	**< 0.001**
Tongue: No Intervention / Chew B	15.0	6.6	34.1	**< 0.001**
Tongue: No Intervention / Tooth brushing	2.3	1.0	5.2	0.055
Tongue: No Intervention / Chew A	2.2	1.0	4.9	**0.049**

Fifty bacterial colonies were randomly selected to determine the taxonomy of VSC-producing bacteria present in plaque and tongue scrape samples. Following purification and 16S sequencing taxonomy was determined for 40 colonies (< 98% similarity) (Table 4). The dominant bacterial species that were identified as black pigmenting VSC-producing bacteria were from the genera *Fusobacterium* and *Peptostreptococcus*. The dominant bacterial taxa that were identified as other coloured colonies, i.e., non-VSC-producing, were from the genera *Actinomyces* and *Streptococcus*.

**Table 4:** Taxonomic identifiers assigned to 40 bacterial isolates identified via 16S sequencing from OHO-C agar

Strain ID number	Location	Original colony colour	16S Sequencing result	Identities %
7367	Plaque	Black	*Actinomyces canis* | COT-409 | Clone 5 U29 | KF030202 | 1 |	99.9
7374	Plaque	Black	*Peptostreptococcus* sp. | COT-033 | Clone OD020 | JN713198 | 101 |	99.7
7375	Plaque	Black	*Fusobacterium canifelinum* | COT-188 | Clone QD074 | JN713355 | 13 |	99.8
7391	Plaque	Black	*Fusobacterium canifelinum* | COT-188 | Clone QD074 | JN713355 | 13 |	99.7
7396	Plaque	Black	*Peptostreptococcaceae* XI [G-2] sp. | COT-047 | Clone OD006 | JN713216	100
7398	Plaque	Black	*Leptotrichia* sp. | COT-345 | Clone 1 J034 | JN713514 | 7 |	99.9
7402	Plaque	Black	*Fusobacterium canifelinum* | COT-188 | Clone QD074 | JN713355 | 13 |	99.4
7406	Plaque	Black	*Leptotrichia* sp. | COT-345 | Clone 1 J034 | JN713514 | 7 |	99.9
7414	Plaque	Black	*Porphyromonas macacae* | COT-192 | Clone QD016 | JN713359 | 16 |	99.8
7370	Plaque	White	*Actinomyces canis* | COT-409 | Clone 5 U29 | KF030202 | 1 |	99.8
7379	Plaque	White	*Porphyromonas macacae* | COT-192 | Clone QD016 | JN713359 | 16 |	99.9
7383	Plaque	White	*Streptococcus minor* | COT-116 | Clone OI055 | JN713284 | 17 |	99.9
7385	Plaque	White	*Porphyromonas macacae* | COT-192 | Clone QD016 | JN713359 | 16 |	99.8
7387	Plaque	White	*Actinomyces canis* | COT-409 | Clone 5 U29 | KF030202 | 1 |	99.8
7400	Plaque	White	*Pasteurella dagmatis* | COT-092 | Clone OE001 | JN713256 | 25 |	100
7409	Plaque	White	*Eikenella* sp. | COT-049 | Clone OB066 | JN713218 | 3 |	99.2
7416	Plaque	White	*Streptococcus minor* | COT-116 | Clone OI055 | JN713284 | 17 |	99.9
7366	Tongue	Black	*Porphyromonas cangingivalis* | COT-109 | Clone QC021 | JN713277 | 203	99.4
7372	Tongue	Black	*Tannerella forsythia* | COT-023 | Clone OB071 | JN713185 | 27 |	99.9
7373	Tongue	Black	*Actinomyces canis* | COT-409 | Clone 5 U29 | KF030202 | 1 |	99.8
7376	Tongue	Black	*Peptostreptococcus* sp. | COT-033 | Clone OD020 | JN713198 | 101 |	99.7
7378	Tongue	Black	*Streptococcus minor* | COT-116 | Clone OI055 | JN713284 | 17 |	99.4
7381	Tongue	Black	*Fusobacterium canifelinum* | COT-188 | Clone QD074 | JN713355 | 13 |	99.9
7386	Tongue	Black	*Peptostreptococcus* sp. | COT-033 | Clone OD020 | JN713198 | 101 |	99.7
7390	Tongue	Black	*Bacteroides heparinolyticus* | COT-310 | Clone 1A034 | JN713478 | 1 |	98.5
7393	Tongue	Black	*Bacteroides heparinolyticus* | COT-310 | Clone 1A034 | JN713478 | 1 |	98
7397	Tongue	Black	*Fusobacterium canifelinum* | COT-188 | Clone QD074 | JN713355 | 13 |	99.7
7403	Tongue	Black	*Fusobacterium canifelinum* | COT-188 | Clone QD074 | JN713355 | 13 |	98.9
7407	Tongue	Black	*Bacteroides heparinolyticus* | COT-310 | Clone 1A034 | JN713478 | 1 |	99.5
7411	Tongue	Black	*Fusobacterium* sp. | COT-236 | Clone QE025 | JN713401 | 4 |	99.4
7415	Tongue	Black	*Porphyromonas macacae* | COT-192 | Clone QD016 | JN713359 | 16 |	99.8
7369	Tongue	White	*Actinomyces canis* | COT-409 | Clone 5 U29 | KF030202 | 1 |	99.8
7377	Tongue	White	*Actinomyces canis* | COT-409 | Clone 5 U29 | KF030202 | 1 |	99.8
7384	Tongue	White	*Actinomyces canis* | COT-409 | Clone 5 U29 | KF030202 | 1 |	100
7388	Tongue	White	*Actinomyces bowdenii* | COT-413 | Strain OH2481 | KF030209 | 0 |	99.1
7392	Tongue	White	*Actinomyces bowdenii* | COT-413 | Strain OH2481 | KF030209 | 0 |	99.8
7394	Tongue	White	*Actinomyces bowdenii* | COT-413 | Strain OH2481 | KF030209 | 0 |	99.4
7401	Tongue	White	*Actinomyces bowdenii* | COT-413 | Strain OH2481 | KF030209 | 0 |	98.8
7408	Tongue	White	*Pasteurellaceae* [G-2] sp. | COT-080 | Clone OC053 | JN713243 | 97 |	99.9
7417	Tongue	White	*Streptococcus minor* | COT-116 | Clone OI055 | JN713284 | 17 |	99.9

## Discussion

The study presented here investigated the efficacy of dental interventions via quantification of VSC levels, specifically H_2_S, CH_3_SH, and (CH_3_)_2_S from canine breath. In addition, the potential for a correlation with bacteria capable of producing VSCs was explored. Oral malodour can be evaluated or measured by a variety of techniques. Many earlier studies have successfully used the Halimeter^®^ to measure VSCs produced in breath, however the approach has been critiqued for being more sensitive to H_2_S than CH_3_SH and almost insensitive to (CH_3_)_2_S [34]. The OralChroma™ has previously been shown to be effective at measuring oral malodour levels in dogs [3] and was therefore selected for use in this study. It is a highly sensitive portable gas chromatograph machine developed for use with human samples which can measure all three gases H_2_S, CH_3_SH, (CH_3_)_2_S independently, and provides quantitative real-time values.

The methodology adopted within this study included cysteine induction to enhance the levels of detectable VSCs. Pre-treatment levels of VSCs were low and considered near to the minimum level of detection for the OralChroma™ likely because the animals used in the study had a good level of oral health due to receiving regular dental care. Following induction with cysteine, post treatment values showed that when two different oral care chews (chew A and chew B) were fed to dogs, the VSC levels for H_2_S and CH_3_SH detected were reduced, compared to the no intervention. In addition, chew B significantly decreased the level of H_2_S and CH_3_SH detection when compared with tooth brushing and no intervention control.

In agreement with reduced VSC levels detected in breath samples, bacterial loads from plaque and tongue samples were significantly lower when dogs had consumed chew B compared to the no intervention controls. Both tooth brushing and chew A also resulted in a significantly lower bacterial number in plaque samples. Chew A has a hard texture and is thought to have a similar action to tooth brushing, only interacting and removing plaque from one side of the tooth and not disturbing the bacteria on the tongue. Chew B has a porous texture that allows the chew to flex around the teeth aiding plaque removal down to the gum line. Physical removal of plaque bacteria can also be achieved by tooth brushing, using gentle abrasion applied to the tooth surface to remove the build-up of plaque. Tooth brushing is highly effective at removing bacteria from tooth surfaces, however tooth brushing in dogs is generally only applied to the outside of the dental arch (buccal side); the lingual side of the teeth and the tongue are not brushed. The results presented here suggest malodour-causing bacteria are not limited solely to the buccal side tooth surface; the tongue also contains a large number of these bacteria. This is supported by human studies where numerous ecological niches have been identified that harbour malodourous bacteria, namely the tooth surface, gingival sulcus and saliva, however the tongue has been described as the most important source of the peptides and mucins which are fermented by bacteria to produce oral malodour [35].

In human studies, the most common organisms identified as VSC-producing are Gram-negative species and proteolytic obligate anaerobes that mainly reside in the tongue coating and periodontal pockets. *Fusobacterium*, *Prevotella, Treponema*, and *Porphyromonas* sp have been found to be associated with the intensity of mouth odour [25, 36–38]. In addition, the presence of specific periodontal pathogens belonging to the genera *Porphyromonas*, *Prevotella*, *Actinobacillus* and *Fusobacterium* have been noted [35]. In this canine study, 16S sequencing revealed 19 operational taxonomic units from 50 samples, representing 10 genera, dominated by *Actinomyces* (27.5%), *Fusobacteria* (17.5%) and *Porphyromonas* (12.5%). The dominant bacterial species that were identified as black pigmenting VSC-producing were from the genera *Fusobacterium* and *Porphyromonas*, a finding comparable to human studies. To gain a deeper understanding of the microbiota present and explore the key species which drive malodour, additional sampling from other oral sites such as lingual surface, buccal mucosa, saliva and the use of next generation sequencing platforms would be advantageous.

In addition to the most common sites of malodour production (tongue, interdental, and subgingival areas), saliva is thought to play a significant role in the generation of VSCs. In humans, VSC levels during the day are inversely related to salivary flow. Salivary flow is at its lowest overnight due to fasting and insufficient water intake, leading to an increase in odour intensity [36]. Conversely, mastication increases saliva flow, with simultaneous flushing of the oral cavity and reduction in malodour. Within our study, we measured the time taken to consume the oral care chews to understand if mastication contributes to the differences observed in VSCs detected. Chew B took the longest to consume, averaging 12 mins. In comparison, the timeframe for the consumption of the other chew was on average 3 minutes. A trend in greater reduction in H_2_S with longer chewing of chew B was observed; however, the correlation was not found to be significant. It is also assumed that the duration of consumption of chew B is one of the factors which causes the observed reduction in numbers of VSC-producing bacteria, due to the chew being in contact with the teeth for longer.

In addition to mechanical removal, the reduction of malodour may be aided using active chemical reagents. Oral care chews A and B both contain the active ingredients zinc sulphate and sodium tripolyphosphate. The primary action of these components is to prevent calculus formation on the tooth surface [36, 39, 40]. There is additional evidence which has shown these active ingredients can inhibit human oral pathogens [41, 42]. Zinc ions have an inhibitory effect on oral malodour, involving two mechanisms of direct binding with gaseous H_2_S and suppressing the growth of VSC-producing oral bacteria [43]. In a study investigating the inhibitory activities of zinc ions on the growth of nine oral bacterial strains, six related to VSC production, *Fusobacterium nucleatum* was found to be the most sensitive species tested [43]. The active agent sodium tripolyphosphate binds with calcium in saliva, making it unavailable for the formation of tartar. Polyphosphates, particularly tripolyphosphates, possess antimicrobial activity and have been shown to inhibit Gram-positive bacteria [44]. They were shown in in vitro cultures to be effective in inhibiting growth of the human oral pathogenic bacteria *Prevotella intermedia*, *Porphyromonas gingivalis* and *Fusobacterium nucleatum* [42]. An additional in vitro study showed sodium tripolyphosphate was inhibitory against *Porphyromonas gulae, Porphyromonas cansulci* and *Porphyromonas cangingivalis* species associated with periodontitis of companion animals [45]. In the present study, *Fusobacterium* sp. and *Porphyromonas* sp. were the dominant VSC-producing species identified from both plaque and tongue samples from the canine oral cavity. We can therefore infer that the active reagents in the chews would be effective against these species specifically and support malodour management alongside physical plaque removal.

Reduction of plaque and calculus levels are common claims for canine dental products with several studies reporting data to support the efficacy of specific canine dental chews on improved oral health [31–33, 46]. In a recent study, Ruparell et al. [19] reported that supplementation of diet with a daily oral care chew increased the proportion of health-associated bacteria over bacteria associated with periodontal disease in canine supragingival plaque. Feeding of dental chews can also impact malodour, providing additional oral health benefits to the dog. Hence, oral care interventions such as dental products, which reduce bacterial plaque accumulation and consequently oral malodour, can support in delivering long term benefits to the health of dogs.

The insights generated here support the opportunity for follow on studies which could investigate the relative abundance of key bacterial species driving oral malodour, and their associations with health, gingivitis and periodontitis disease. This would build fundamental understanding regarding the role of VSCs produced by these bacteria and could lead to knowledge relating to their toxicity in tissues and an associated role in the pathogenesis of periodontal disease. The outcomes may lead to potential targets for antimicrobial therapy and the prospect for using VSCs as biomarkers for periodontal disease progression.

## Conclusion

Microbial plaque is an aetiological agent for gingivitis, periodontitis and oral malodour; therefore, controlling plaque plays an important role in the maintenance of good oral health. Mechanical removal of plaque by tooth brushing is the most common tool used in human dental hygiene. However, tooth brushing by pet owners is not well practiced, and even if tooth brushing is performed competently, it does not usually involve brushing other oral surfaces such as the tongue, upon which significant numbers of malodour-generating bacteria can be present. Thus, the use of oral care products which are designed to remove bacteria from multiple oral sites which are not usually accessed by tooth brushing may be an effective means of reducing oral malodour and maintaining periodontal health.

## Methods

### Study cohort and ethics statement

Dogs housed at the WALTHAM Petcare Science Institute (Melton Mowbray, Leicestershire, UK) were recruited to participate in the study. The WALTHAM Animal Welfare and Ethical Review Body approved the study which was run under licensed authority in accordance with the UK Animals (Scientific Procedures) Act 1986.

A power analysis was performed using data from a pilot study containing 15 dogs, aiming for 80% power to determine the sample size to detect a 10-fold change, determining the requirement for 13 adult dogs for this study. Fourteen medium-breed size dogs were recruited to the study to allow for single dropout from the cohort, comprising 5 male and 4 female Petit Basset Griffon Vendéen (PBGV) and 5 female Beagle dogs; all were neutered adults. The average age was 3.9 years (between 2.2 and 7.4 years) and bodyweights ranged between 13.4 and 16.7 kg. Dogs were pair-housed in environmentally enriched kennels and provided with comprehensive dog-dog and dog-human socialisation programmes adjusted to the needs of individual dogs. As part of their normal husbandry routine, dogs had full mouth checks during their daily tooth brushing sessions to assess the health of teeth and gums before the start of the study.

Dogs were routinely fed commercially available, nutritionally complete and balanced diets, which conformed to the National Research Council Nutrient Guidelines 2006 (National Research Council 2006). Dogs were fed according to their individual energy requirement to maintain bodyweight. Daily calorie intake was reduced on the corresponding study days to account for the consumption of an oral chew.

### Sampling strategy

All dogs were exposed to the oral interventions in a randomised crossover balanced Latin square design. The interventions comprised either the consumption of one of two oral care chews (A – prototype or B – Pedigree^®^ Dentastix™ Advanced, with a porous sponge like texture), or a minimum 2 min tooth brushing of the outer buccal tooth surfaces. The control groups received no intervention. Each of the four treatments were repeated three times across the 14-week study, with dogs undergoing each event once every 4 weeks. After all samples (breath, plaque and tongue swabs), had been taken the dogs had their teeth brushed before returning to their pen. This was to control plaque accumulation between sample collections.

### Breath sample collection

The detection and measurement of individual VSCs in breath samples was measured using a OralChroma™ device (Nissha Inc., Japan) calibrated with individual gases by the supplier prior to the start of the study. Standard anesthesia masks correct for the breed sizes and skull morphology, with a piece of Saint-Gobain Clear Tygon™ plastic tubing threaded through the end port were prepared. Dogs were habituated to the masks and voluntarily placed their heads into the masks and place their lips around the tube. When dogs had established a natural pattern of breathing, usually within 30 seconds, and a seal had been formed around the tubing, a 5 ml syringe was drawn to collect air from the oral headspace. As per the OralChroma™ manufacturer’s instructions, 4 ml was expelled, and 1 ml was then injected into the device for analysis. The headspace concentrations (ppb) of three volatile sulphur compounds (VSCs) – hydrogen sulphide (H_2_S), methyl mercaptan (CH_3_SH) and dimethyl sulphide ((CH_3_)_2_S) was recorded following comparison to standard curves for each gas.

Malodour was induced by sulphuric amino acids through the consumption of one 15 ml cube of 1% gelatine gel (6 mM cysteine and 6 mM methionine) and 90 ml solution (6 mM cysteine and 4 mM methionine). The former was designed to melt in the mouth and around the buccal surfaces to induce malodour, and the latter to coat the tongue whilst the dog lapped at the amino acid solution. The concentrations of 6 mM cysteine and 6 mM methionine were determined through pilot feasibility assessments where concentrations were tested up to 12 mM. No additional increase in VSCs was identified above 6 mM when concentrations of the cysteine and methionine were increased.

Breath samples were collected at two time points for each intervention: immediately after the intervention (VSC pre-induction) and immediately after VSC induction by sulphuric amino acids (VSC post-induction). There was an interval of 20 minutes between the VSC pre-induction measurement and amino acid induction to ensure the oral cavity was equilibrated for oxygen levels [23] Timings were recorded for the duration of the head within the mask, gel and solution consumption, tooth brushing and oral care chew consumption.

### Supragingival plaque and tongue scrape sample collection

Supragingival plaque and tongue scrape samples were collected from the dogs after pre- and post-induction breath samples had been collected. Plaque was collected using sterile microbiological loops (Thermo Scientific, Loughborough, UK) from the buccal sides of the 106/206, 107/207 and 108/208 teeth (2nd, 3rd, and 4th premolars, respectively) from the upper jaw and 307/407 and 308/408 teeth (3rd and 4th premolars) from the lower jaw. Posterior tongue dorsum samples were collected through gentle scrapes from the tongue surface using a cytology brush (Medical Packaging Corporation, USA). Loops and brushes were immediately immersed into individual aliquots of 300 μl Phosphate Buffered Saline (PBS) buffer and processed as described below within 30 minutes of collection.

Samples were serially diluted in PBS from neat to 10^7^ onto Oral Hydrogen Sulfide Organisms (OHO-C) agar with Lead Acetate (Anaerobe Systems, USA) prior to anaerobic incubation at 38 °C for 7 days (MACS MG 1000 Anaerobic Workstations 80% N_2_/10% CO_2_/10% H_2_; Don Whitley Scientific Limited, Bingley, UK). OHO-C agar supports the growth of oral bacteria which appear as white colonies on the agar surface. However, those bacteria which produce VSCs appear as black colonies due to precipitates of lead sulphides on the agar surface. Colony counts were calculated for both black pigmented bacteria and total bacteria (white and black colonies combined).

Bacterial species identification was performed through culturing on the OHO-C agar. All samples from three dogs were purified and identified by 16S Sanger Sequencing (performed by Source Bioscience Plc, Nottingham, UK) performing BLAST on bacterial sequences using sources NCBI websites.

### Statistical analyses

For each VSC, a linear mixed effects model was fit for the log_10_ (pre induction abundance+ 1) and the log_10_ (post induction abundance+ 1) against intervention as the fixed effect and dog as the random effect. The estimated average abundance for each intervention was reported with 95% confidence intervals for each VSC. Dunnett’s comparisons were then made from no intervention to all other interventions, and the subsequent fold changes in means are reported with 95% confidence intervals and *p*-values.

For the recorded timings from the study, correlation with the VSC abundance was investigated. The duration of intervention was plotted against VSC pre- and post-induction abundance. For each treatment, the Pearson correlation between VSC and duration of intervention is estimated.

For the microbiological colony data, models were fit for log_10_ (total colony CFU per ml + 1), log_10_ (black colony CFU per ml + 1), and % black colonies. Each took the form of a linear mixed effects model with fixed effects of sample type (plaque or tongue) and intervention with their interaction and random effects of plate batch nested within cycle within dog. Estimated means and 95% confidence intervals were extracted for each combination of sample type and intervention. Comparisons were performed between sample types at each intervention level, and from the no intervention to all other interventions within each sample type.

For all analyses a 5% family-wise corrected error rate was used for the comparisons. Statistical analyses were performed using the statistical software R. The packages used were: ggplot2 (v3.3.2) and ggsignif (v0.6.0) for graphical representations, lme4 (v1.1-21) and multcomp (v1.4-12) for linear mixed models and multiple comparisons.

## Data Availability

The datasets generated and analysed during the current study are available in the GenBank repository, accession numbers as follows; ON222746, ON222747, ON222748, ON222749, ON222750, ON222751, ON222752, ON222753, ON222754, ON222755, ON222756, ON222757, ON222758, ON222759, ON222760, ON222761, ON222762, ON222763, ON222764, ON222765, ON222766, ON222767, ON212014, ON212015, ON212016, ON212017, ON212018, ON212019, ON212020, ON212021, ON212022, ON212023, ON212024, ON212025, ON212026, ON212027, ON212028, ON212029, ON212030, ON222793.

## Abbreviations

VSCs: Volatile sulphur compounds
PBGV: Petit Basset Griffon Vendéen
CH_3_SH: Methyl mercaptan
H_2_S: Hydrogen sulphide
(CH_3_)_2_S: Dimethyl sulphide
OHO-C: Oral Hydrogen Sulfide Organisms agar
ppb: parts per billion
